# E4orf1 Suppresses *E1B*-Deleted Adenovirus Vaccine-Induced Immune Responses

**DOI:** 10.3390/vaccines10020295

**Published:** 2022-02-15

**Authors:** Kotou Sangare, Sabrina Helmold Hait, Madison Moore, Christopher Hogge, Tanya Hoang, Mohammad Arif Rahman, David J. Venzon, Celia LaBranche, David Montefiori, Marjorie Robert-Guroff, Michael A. Thomas

**Affiliations:** 1 Department of Biology, Howard University, Washington, DC 20059, USA; ksangare09@gmail.com (K.S.); mmadison_mmoore@yahoo.com (M.M.); 2 Section on Immune Biology of Retroviral Infection, Vaccine Branch, National Cancer Institute, National Institutes of Health, Bethesda, MD 20892, USA; helmoldhaits@niaid.nih.gov (S.H.H.); cjhogge127@gmail.com (C.H.); hoangt@dc37a.nci.nih.gov (T.H.); mohammadarif.rahman@nih.gov (M.A.R.); guroffm@dc37a.nci.nih.gov (M.R.-G.); 3 Biostatistics and Data Management Section, National Cancer Institute, National Institutes of Health, Bethesda, MD 20892, USA; venzond@mail.nih.gov; 4 Duke University Medical Center, Durham, NC 27710, USA; celia.labranche@duke.edu (C.L.); david.montefiori@duke.edu (D.M.)

**Keywords:** adenovirus, HIV, vaccine, nonhuman primates, early region 4 open reading frame 1 (*E4orf1)*, memory B-cells, antibody, cytokine genes, antibody neutralization, ADCC

## Abstract

As demonstrated by the recent COVID pandemic, vaccines can reduce the burden arising from infectious agents. Adenoviruses (Ads) with deletion of the early region 1B55K (ΔE1B Ad) are currently being explored for use in vaccine delivery. Δ*E1B* Ads are different from Ads with deletions in early region 1 and early region 3 (ΔE1/E3) used in most Ad vaccine vectors in that they contain the Ad early region 1A (E1A), and therefore the ability to replicate. Common to almost all Ads that are being explored for clinical use is the Ad early region 4 (E4). Among the E4 genes is open reading frame 1 (E4orf1), which mediates signals through the PI3-kinase/Akt pathway that is known to modulate immune responses. This suggests that E4orf1 might also modulate immune responses, although it has remained unexplored in Δ*E1B* Ad. Here, we show that cells infected with an *E1B55K* and *E4orf1*-deleted (Δ*E4^1^*) Ad exhibited reduced levels of phosphorylated Akt (Ser473 and Thr308)) and expressed different intrinsic innate immune cytokines from those induced in cells infected with an *E4orf1*-containing, Δ*E1B* parental Ad that exhibited elevated levels of phosphorylated Akt. Rhesus macaques immunized with a Δ*E4^1^* Ad that expressed rhFLSC (HIV-1_BaL_ gp120 linked to rhesus CD4 D1 and D2), exhibited higher levels of rhFLSC-specific interferon γ-producing memory T-cells, higher titers of rhFLSC-specific IgG1 binding antibody in serum, and antibodies able to mediate antibody-dependent cellular cytotoxicity (ADCC) with greater killing capacity than the Δ*E1B* Ad. Therefore, E4orf1, perhaps by acting through the PI3-kinase/Akt pathway, limits intrinsic innate and system-wide adaptive immune responses that are important for improved Δ*E1B* Ad-based vaccines.

## 1. Introduction

Of the COVID-19 vaccines available worldwide, 6 of 8 are based on adenovirus (Ad) vectors [1] deleted in early region 1 and early region 3 (Δ*E1/E3* Ad). Besides COVID, Δ*E1/E3* Ads are also being developed for use in vaccine delivery against human immunodeficiency virus type 1 (HIV-1) and other infectious pathogens [2,3]. While popular, the Δ*E1/E3* form of Ad is not as immunogenic as Ad that contains the early region 1 (*E1*). In fact, in one study a phenotypic wild type Δ*E3* Ad HIV vaccine vector was shown to induce a higher frequency of HIV-specific interferon gamma-secreting and proliferating T-cells, higher anti-HIV binding and neutralizing antibody titers, and better induction of ADCC than the Δ*E1/E3* Ad [4]. The Ad *E1* consists of early region 1A (*E1A*), and early region 1B (*E1B55K* and *E1B19K*). E1A and the products of the E1B genes maintain an antagonistic relationship. For example, the actions of E1A sensitize cells to natural killer cell and macrophage-mediated lysis [5]. For its part, E1B55K acts to lessen the effects of E1A by suppressing the expression of immune response genes [6] and by countering the effect of the virus-induced interferon responses [7]. Besides E1B55K, products of the Ad early region 4 (*E4*) are reported to limit the ability of Ad-infected cells to mount an innate immune response [8,9,10]. Accordingly, we recently reported that an Ad deleted in *E1B55K* and *E4* open reading frames 1 to 4 (*E4orf1* to *E4orf4*) in which we replaced the E3 with HIV-1_BaL_ gp120 linked to rhesus macaque CD4 D1 and D2 (rhFLSC) [11], referred to here as Δ*E4^1−4^* Ad*,* promoted higher levels of rhFLSC-specific binding antibodies in immunized mice and rhesus macaques than either the Δ*E3* or the Δ*E1B* Ads [12]. The results suggest that Ad *E4orf1, E4orf2, E4orf3* and/or *E4orf4* act to limit immune responses.

The Ad E4orf1 is reported to activate signals through the PI3-kinase/Akt pathway [13]. In addition to cell survival, the PI3-kinase/Akt pathway also mediates immune responses [14] and is implicated in the phosphorylation of NF-κB [15] which is best known for its involvement in the induction of innate cytokine genes important for stimulating both innate and adaptive arms of the immune system [16]. In another article [17], we showed that E4orf1 signals induce NF-κB. To evaluate the possibility that E4orf1 could mediate immune responses, we created a Δ*E1B* Ad in which we deleted E4orf1 and replaced the E3 with rhFLSC [11], similarly to what we have done before [12,18,19,20]. We termed this new Ad Δ*E4^1^*.

Here we report that, in cell-based experiments, the Δ*E4^1^* Ad failed to induce the phosphorylation of Akt and promoted the expression of intrinsic innate cytokine genes that were different from those expressed in cells infected with the *E4orf1*-containing, Δ*E1B*-parental Ad that induced elevated levels of phosphorylated Akt (Ser473 and Thr308). Rhesus macaques that were immunized with the Δ*E4^1^* Ad exhibited higher levels of HIV-specific IFNγ-secreting memory T-cells, higher serum levels of HIV-specific IgG1, and higher levels of ADCC functional antibodies than rhesus macaques that were immunized with the Δ*E1B* Ad. Thus, E4orf1 limits intrinsic innate and system-wide adaptive immune responses that are important for improved Δ*E1B* Ad-based vaccines.

## 2. Materials and Methods

### 2.1. Viruses

The Δ*E3* virus, Ad5rhFLSC, has been described elsewhere [20]. In brief, 2800 base pairs in the *E3* region of Adhr404 [21] were replaced with rhFLSC. The mutant virus *dl*1520 contains an 827-bp deletion in the region encoding the 55kDa protein [22]. The *Ad5hr*Δ*E1B55K/*Δ*E3*rhFLSC (Δ*E1B*), *Ad5hr*Δ*E1B55K/*Δ*E3/*Δ*E4orf1-4*rhFLSC (Δ*E4^1−4^*) and *Ad5hr*Δ*E1B55K/*Δ*E3/*Δ*E4orf1*rhFLSC (Δ*E4^1^*) viruses were created by recombining Spe1 digested dl1520 DNA with BamHI digested pBRAd5hrΔE3 (TPL-rhFLSC-pA) plasmid in which we deleted the coding regions for E4orf1 or E4orf1–4. The resulting viral DNAs were screened by PCR for the presence or absence of the *E1B55K* and the *E4* genes. The viruses were purified and concentrations determined as described in [13] by plaque assays.

### 2.2. Cell Culture

All cell lines were obtained from the American Type Culture Collection. We used the human cervical carcinoma-derived HeLa cell line (ATCC^®^ CCL-2™) because most of what is known about Ad is in the context of this cell line. We used the human lung A549 cell line (ATCC^®^ CCL-185) as Ad is known to target the upper respiratory airway. The human embryonic kidney-derived 293 cell line was used because it supports the growth of mutant Ad. All the cells were maintained in Dulbecco’s modified Eagle’s medium (DMEM 1x) (ref# 11965-092) supplemented with 10% fetal bovine serum (ref# 16000-044) and 1% penicillin streptomycin (ref# 15140-122). Cell culture media and supplements were obtained from Life Technologies (Carlsbad, CA). Cell lines were maintained at 37 °C in a humidified atmosphere with 5% CO_2_ as previously described [20].

### 2.3. Gel Electrophoresis and Western Blot

Gel electrophoresis and western blotting were performed as described [20]. Briefly, cells were infected at a MOI of 5–50. After 24, 48, and 72 h, 50–100 μL of growth media was collected and diluted in 1X SDS Gel loading Dye plus 3% BME. Odyssey^®^ Protein Molecular Weight Marker (Li-COR) along with equal amounts of samples/lysate were run on 4–20% SDS-polyacrylamide gels and transferred to nitrocellulose membranes using the iBlot Western Blot System (Life Technologies, Carlsbad, CA, USA). Blots were blocked in PBS-0.02% Tween 20 containing 5% milk for 2 h and exposed to a 10% milk buffer containing one of the following primary antibodies at 4° overnight or for 1 h at room temperature: anti-beta tubulin (Thermo Fisher Scientific, Waltham, MA, USA, Cat#PA1-16947); rabbit anti-HIV-1_Ba-L_ (clade B) gp120 (Cat# 5420, Advanced Bioscience Laboratories, Inc. (ABL), Rockville, MD, USA); goat anti-hCD4 IgG (Cat# AF-379-NA, R&D Systems); mouse anti-DNA binding protein (DBP) (gift from David Ornelles, Wake Forest University, NC, USA); and rabbit polyclonal anti-Ad type 5 (Cat# Ab6982, Abcam). Subsequently, the blots were washed and exposed to an HRP conjugated secondary antibody: goat anti-mouse IgG (Cat# 31439, Invitrogen), goat-anti rabbit IgG (H + L) (Cat# 31460, Invitrogen, Waltham, MA, USA), or mouse-anti goat IgG (H + L) (Cat# 31400, Invitrogen) as dictated by the primary isotype. Chemiluminescent detection was performed using SuperSignal West Pico Chemiluminescent Substrate (Thermo Fisher Scientific, Waltham, MA, USA) or LumiGLO Chemiluminescent Substrate System (KPL).

### 2.4. DNA Extraction and PCR

DNA was extracted using the Qiagen DNA blood Mini kit (Cat# 51106) following the manufacturer’s procedure. The purity and quantity of all extracted DNA were determined using a Nano Drop spectrophotometer (Thermo Fisher Scientific, Waltham, MA, USA). The PCR assays were performed as described [12,20] with minor modifications. Briefly, test sample DNA (15 to 100 ng) was added to the Dream Taq Green master mix (Thermo Fisher Scientific, Waltham, MA, USA, Cat # K1081). Reverse and forward primers for Ad fiber and PCR-quality water (Thermo Fisher) were added to reach a final volume of 25 μL. A negative control (no template DNA) was included in each run. PCR amplification was performed using a DNA Thermocycler (SimpliAmp Thermal Cycler, Life Technologies), as follows: 2 min at 94 °C, then 25 cycles of 94 °C, 1 min; 55 °C, 30 s; 72 °C, 2 min, and a final 5 min extension period at 72 °C. As a control of the PCR product size, 12 μL of PCR product was loaded on a 1% 1X TAE buffer-containing agarose gel, stained with 0.3 μg/mL ethidium bromide, and photographed with a LI-COR imaging system. The molecular weight of the PCR products was determined by comparison to Gene Ruler 1 kb plus DNA ladder (Thermo Fisher Scientific, Waltham, MA, USA, Cat# SM1333).

### 2.5. Determination of Virus Yield

The viral progeny was determined by seeding 5 × 10^5^ A549 or HeLa cells in 6 well plates that were infected afterwards with the Ad vectors at two different concentrations (5 and 50 MOI). Media of infected cells were collected at three different time points (24, 48 and 72 h post infection), centrifuged, and the supernatant was used to determine the viral yield by plaque assay. Monolayer HEK-293 cells were infected with serially diluted supernatants. After removal of the infected solution, cells were washed one time with 1x PBS and 2 mL of layer medium (half 2% Low Melting Point agarose and half Modified Eagle Medium (2x) with no phenol red and 4% FBS) was added at day zero, day five and at day 8 with 0.1% neutral red solution. Plates were incubated for 10 to 14 days, and the plaques were counted. Numbers were plotted and analyzed with GraphPad Prism software version 9 (GraphPad Software).

### 2.6. RNA Extraction and Real Time Quantitative RT-PCR

To assess innate cytokine network gene expression, RNA extraction and RT-PCR were performed as described [12]. Cells were either not infected (mock) or infected with Δ*E1B* or the Δ*E4^1^* virus at a MOI of 50 PFU/cell for 48 h. Total RNA was isolated with RNAzol^®^ RT (Sigma) based on the manufacturer’s recommended protocol. RNA concentrations were determined using a Nano Drop spectrophotometer (Thermo Fisher Scientific, Waltham, MA, USA). The RNA was reverse transcribed to cDNA with a Quantitect^®^ reverse Transcription Kit (QIAGEN, Germantown, MD, USA) according to the manufacturer’s instructions. Briefly, samples were treated with 10 U DNase (Pharmacia) and incubated for 2 min at 42 °C for genomic DNA elimination, followed by 30 min for cDNA synthesis and 95 °C for 3 min to inactivate Quantiscript Reverse Transcriptase. Gene expression was assayed using a TaqMan^®^ Array Human Cytokine Network 96-well FAST Plate (Thermo Fisher Scientific, Waltham, MA, USA, Cat # 4418769). The TaqMan^®^ Array Plate contains 28 assays for genes associated with pro- and anti-inflammatory cytokines and four assays for candidate endogenous control genes. The quantitative real-time PCR amplification was performed using QuantStudio™ 6 Flex Real-Time PCR System (Applied Biosystems^®^, Waltham, MA, USA). The PCR program used consisted of sample holding at 50 °C for 2 min and then 95 °C for 10 min, followed by 40 cycles, each consisting of 95 °C for 15 s, 56 °C for 15 s, and 62 °C for 30 s. Assays were performed two times in triplicate. The highest cycle number, 40, was used for all undetermined values. Mock infected cells were used as negative control. The ΔΔct number of the control cells was subtracted from the experiment for each target. All data were normalized with 18S ribosomal RNA. The 2^-ΔΔct^ fold number for each experiment was determined and converted into Log2. Calculations and statistical analysis of the results were performed using GraphPad Prism software version 9 (GraphPad Software).

Genomic DNA contamination was determined by performing RT-qPCR with total RNA or same sample cDNA as the template for a segment of Ad fiber gene as described in the DNA extraction and PCR step. No PCR product appearing on the total RNA gel showed that no genomic DNA contaminated the total RNA. Product integrity was also evaluated using 0.8% agarose gel electrophoresis and showed no contamination with DNA or degradation of RNA [23,24].

### 2.7. Animal Immunization

A pilot study was conducted in rhesus macaques (*Macaca mulatta*) in which three macaques per group were immunized with rhFLSC-expressing Δ*E3,* Δ*E1B* or Δ*E4^1−4^*Ad5hr [12]. We brought the total to 5 macaques per group and added a fourth group of 4 macaques that were immunized with Δ*E4^1^* Ad5hr. The animals were housed and maintained at the NCI Animal Facility, (NIH, Bethesda, MD, USA) according to the standards of the American Association for Accreditation of Laboratory Animal Care. All rhesus macaques were negative for SIV, simian retrovirus type D and STLV. The protocol (VB-032) was reviewed and approved by the NCI Animal Care and Use Committee prior to implementation. Animals were immunized at week 0 intranasally and orally and at week 12 intratracheally with the Ad recombinants at a dose of 5 × 10^8^ PFU/route/macaque and boosted intramuscularly with recombinant rhFLSC protein (Profectus BioSciences, Inc., Baltimore, MD), a total of 100 μg/macaque, in 1 mL of 1× phosphate buffered saline (PBS) plus an equivalent volume of Alum adjuvant (Aluminum hydroxide gel cat# vac-alu-250) at weeks 24 and 36.

### 2.8. Tissue Preparation

Rectal tissues were processed as described [25] with minor modification. Briefly, mucosal tissues were collected in R10 (RPMI1640 containing anti-fungal-bacterial solution, L- glutamine and 10% FBS), spun down, and digested in 10 mL of pre-warmed digestive medium (RPMI1640, anti-fungal- bacterial solution, 2-mM L-Glutamine (all Invitrogen) and 2 mg/mL Collagenase (Sigma-Aldrich, St. Louis, MO, USA)) for 45 min at 37 °C with mixing every 5 min. The digested tissues were added to 40 mL of RPMI to stop the collagenase reaction. After spinning down, 10 mL of R10 was added to each sample and passed 5 or more times through a blunt end cannula attached to a 12 G syringe. Liberated cells and tissue debris were passed through a 40 µm cell strainer and the rectal cells were collected into an attached 50mL tube. BM and PBMC were purified by ficoll gradient centrifugation, frozen with 10% FBS in DMSO as previously described [26], and stored in liquid nitrogen until analysis. In addition, serum samples were collected, aliquoted, and stored at −70 °C until they were analyzed. Live cells were counted using trypan blue.

### 2.9. Intracellular Cytokine Staining

Freshly isolated PBMC (2 × 10^6^) were stimulated with pools of HIV_BaL_ gp120 peptides (ABL) and stained as described previously [12,27] with the antibodies listed in Table 1. Approximately 500,000 lymphocytes were acquired for analysis using an LSRII Flow Cytometer. A singlet was applied first, followed by live/dead and then lymphocytic gates. CD3+ T-cells were divided into CD4+ and CD8+ populations, and each population was further subdivided into CD28+ CD95+ central memory (CM) and CD28−CD95+ effector memory (EM) cells. The percentage of cytokine-secreting cells in each memory cell subset was determined following subtraction of the values obtained with non-stimulated samples. Both subsets were summed to give the total memory (TM) T-cell population. Data points that were below the background were given the value 0.001 or 0.002 for graphical representation. Data were analyzed using FlowJo software (FACS Diva).

### 2.10. ELISA Assay

Antibodies in rectal secretions were assayed by enzyme-linked immunosorbent assay (ELISA) as described previously [28]. Rectal secretions were filtered with centrifugal filters (Durapore PVDF 5.0 μm; Merck Millipore, Tullagreen Carrigtwohill, CO, USA) prior to assay. The filtrates were tested for blood contamination using Chemstrips 5OB urine test strips (Roche, Indianapolis, IN, USA) and were not assayed if positive for blood. For binding antibody levels, half area plates were coated with 100 ng/well of rhFLSC for Env-specific IgG and IgA antibody, and 50 µL of 1 µg/mL unconjugated anti-monkey IgG or IgA (AlphaDiagnostic) for total IgG and IgA antibody in sodium-bicarbonate buffer (pH 9.6) overnight at 4 °C. The plates were blocked with 185 µL of 1% BSA in D-PBS for 2 h at room temperature. Rectal swabs were serially diluted in PBS containing 1% BSA. Fifty microliters of serial five-fold dilutions were incubated on the plates for 1 h at 37 °C. The plates were then washed 4 times and 50 µL of HRP-conjugated goat anti-monkey IgG or IgA (AlphaDiagnostic) at 1:10,000 dilution in 1% BSA solution were added. The plates were incubated at room temperature for 1 h, washed 4 times, and TMB substrate (KPL) was used in sequential steps, followed by reading the OD at 450 nm after stopping the reaction with 1M sulfuric acid. For total IgG and IgA antibodies, standards were obtained from the Nonhuman Primate Reagent Resource and goat anti-monkey HRP conjugates were used as detection antibodies at a 1:10,000 dilution. Env-specific IgG and Env-specific IgA standard curves were generated using Env-specific IgG and IgA derived from purified serum IgG and IgA obtained from SIV_mac251_-infected macaques and quantified as described [26]. Rectal swab binding antibodies were reported as ng Env-specific IgG or IgA per ug total IgG or IgA.

Serum-binding antibodies were assayed by ELISA as described previously [20,29]. Ninety-six well plates were coated with 100ng per well of rhFLSC (Profectus BioSciences). The plates were exposed to 1% BSA blocking solution (KPL) for 2 h at room temperature. Serum samples were serially diluted, applied to duplicate wells, and incubated at 37 °C for 1 h. Plates were washed with PBS–Tween, exposed to either peroxidase-conjugated mouse anti-rhesus IgA (pr#-0120 NIH Nonhuman Primate Reagent Resource), or mouse anti-rhesus IgG1 (pr#-7110 NIH Nonhuman Primate Reagent Resource), and incubated for another hour. After washing, the plates were developed with TMB (3, 3′, 5, 5′-tetramethylbenzidine) peroxidase substrate solution. The reaction was stopped by adding 1M H_3_PO_4_ and the plates were read at 450 nm within 30 min on a Biotek ELISA reader (ELx803) and analyzed with Gen5 3.02 software. The endpoint titers were defined as the reciprocal of the serum dilution resulting in two times the OD corresponding to the background value of the plate as the cutoff and were plotted and analyzed with GraphPad Prism.

### 2.11. ADCC Assay

ADCC activity was assessed as previously described [30] using constitutive GFP expressing EGFP-CEM-NKr-CCR5-SNAP cells as targets. Briefly, one million target cells were incubated with 50 μg of BAL gp120 or rhFLSC recombinant protein for 2 h at 37 °C, washed, and labeled with SNAP-Surface Alexa Fluor 647 (New England Biolabs, Ipswich, MA; cat. # S9136S) as recommended by the manufacturer for 30 min at RT. Heat inactivated plasma samples were serially diluted (7 ten-fold dilutions starting at 1:10) and 100 μL were added to a 96-well V-bottom plate (Millipore Sigma). Following this, 5000 gp120-coated target cells (50 μL) and 250,000 human PBMCs (50 μL) as effectors were added to each well to give an effector/target (E/T) ratio of 50:1. The plate was incubated at 37 °C for 2 h followed by two PBS washes. The cells were resuspended in 200 μL of a 2% PBS–paraformaldehyde solution and acquired on an LSRII equipped with a high-throughput system (BD Biosciences, San Jose, CA, USA). Specific killing was measured by loss of GFP from the SNAP-Surface Alexa Fluor 647+ target cells. Target and effector cells cultured in the presence of medium were used as negative controls.

### 2.12. Antibody Neutralization Assay

The ability of serum antibodies from the immunized rhesus macaques to neutralize simian-human immunodeficiency virus SHIVBal-P4 was assessed in TZM- bl cells as described previously [31,32], using murine leukemia virus (MLV)-pseudotyped virus as a negative control. Antibody-neutralizing titers were defined as the reciprocal serum dilution at which there was a 50% reduction in relative luminescence units compared to virus control wells.

### 2.13. Antigen-Specific Memory B-Cell Staining

To investigate the induction of systemic and mucosal rhFLSC-specific memory B-cells, rectal biopsies and BM were collected from each rhesus macaque before immunization (pre) and over the course of immunization. For flow cytometry, 2 × 10^6^ cells/tube were stained as previously described [29] with the antibodies listed in Table 2. rhFLSC was detected using goat anti-hCD4 IgG (Cat# AF-379-NA, R&D Systems) that was conjugated to biotin and APC-streptavidin. After 30 min surface staining at 4 °C, cells were washed in PBS, fixed and permeabilized according to the manufacturer’s instructions using Fix and Perm or a transcription buffer set for IRF-4 (BD Bioscience, San Jose, CA). After washing in permwash solution, intracellular staining was conducted. Subsequently, the cells were washed and resuspended in FACS buffer and kept at 4 °C. Five hundred thousand live cells in the lymphocytic gate were acquired within 24 h on an LSR II (BD Bioscience). Briefly, B-cells were identified by gating on singlets, then lymphocytes, and exclusion of dead cells. Next, CD2+ (NK and T-cells) and CD14^+^ (monocytes) cells were excluded and B-cells were identified as either CD19+ CD20+ and/or CD19+ CD20-. Mucosal class-switched memory B-cells were defined as CD21+ IgD-. BM class-switched memory B-cells were defined as CD27+ IgD-. rhFLSC-specific memory B-cells were identified by gating on class-switched memory B-cells using a biotinylated rhFLSC protein. The percent of rhFLSC-specific memory B-cells was determined by dividing the target events by the total B-cell events. A minimum of 200,000 live B-cells were acquired with FACSDIVA. Analysis was performed in FlowJo, data were exported, and graphs were generated using GraphPad Prism 9. Data points that were lower than the background were given the value 0.01 for graphical representation.

### 2.14. Statistical Analysis

Initial statistics were obtained using Prism v9.0 (GraphPad Software Inc., San Diego, CA, USA) and further confirmed using SAS/STAT software (SAS Institute Inc., Cary, NC, USA). Data were analyzed with paired or unpaired Mann–Whitney T-tests or Wilcoxon signed-rank tests and ANOVA for groups of two or more. Data were considered significant if *p* < 0.05.

## 3. Results

### 3.1. Basic Characteristics of E4 Deleted ΔE1B Ad-Infected Cells

**Figure 1:**
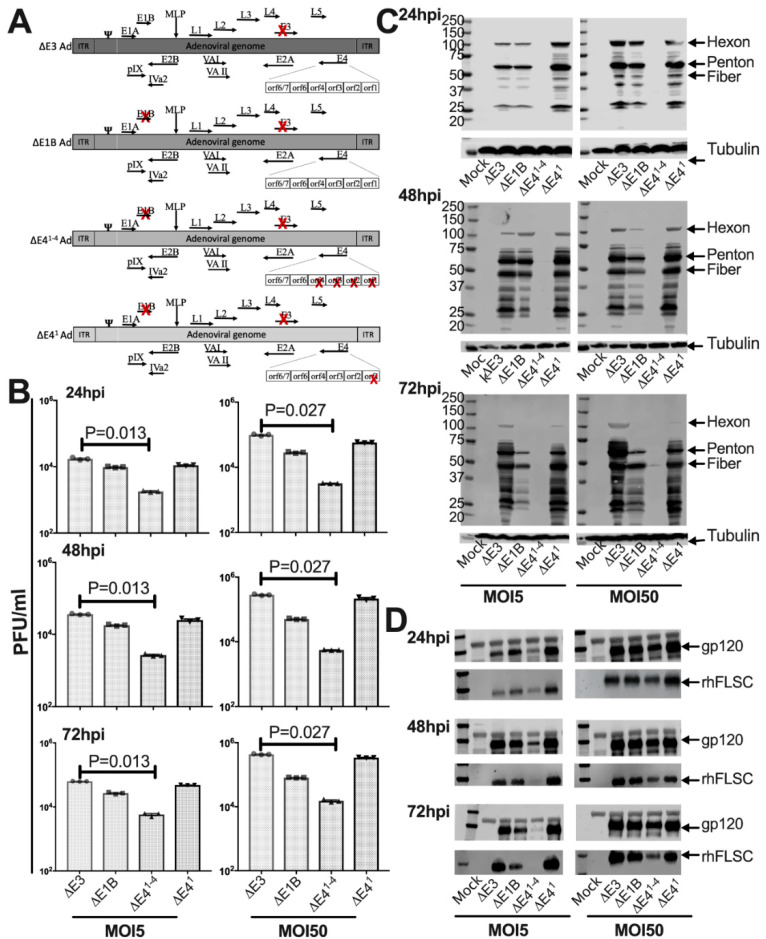
Characterization of Ad5hr HIV-vaccine candidates in infected A549 cells. (**A**) A schematic of the Ads used in this study. A red X marks the deleted genes in each of the Ads. (**B**–**D**) A549 cells were infected at an MOI of 5 or 50 for 24, 48 and 72 h. (**B**) Viral progeny were assessed by plaque assay using HEK-293 cells. Mean values ± SEM were plotted. (**C**) Ad late proteins (hexon, penton and fiber indicated) with tubulin, and (**D**) gp120 and rhFLSC were assessed by western blot. All experiments were repeated at least 3 times. *p* values were obtained using the Mann–Whitney unpaired *t*-test.

In Figure 1A a schematic of the different Ads used in this study is provided. The rhFLSC-expressing Δ*E3,* Δ*E1B*, and Δ*E4^1−4^* Ads were described elsewhere [12]. We also reported on Ad with deletions in *E1B55K* and *E4orf1* before [13]. E4orf1 is an oncogenic protein [33,34,35] that plays a role in Ad late protein production, progeny production [13], lipid metabolism [36], and rates of cell death in infected cells [13]. However, whether E4orf1 affects transgene expression has not been evaluated. We therefore created an Ad deleted in *E1B55K* and *E4orf1* that expressed the HIV transgene, rhFLSC, from the Ad Major Late Promoter (MLP) in place of the Ad E3. We compared progeny yields and levels of early and late proteins, and transgene production in infected cells (Figure 1). In Figure 1B the Δ*E3* Ad-infected cells have the highest levels of viral progeny, followed closely by the Δ*E4^1^* Ad-infected cells, with a reduction seen for Δ*E1B*-infected cells and an even greater reduction for the Δ*E4^1−4^* Ad-infected cells at all times post infection. The progeny levels paralleled levels of the late viral proteins, in that when late protein production was low (Figure 1C), viral progeny yields were low (Figure 1B) as previously suggested [12]. Overall, the patterns of late viral proteins and of progeny yield shown in Figure 1 are similar to those previously published for the Δ*E3,* Δ*E1B*, and the Δ*E4^1−4^* Ad [12] and for *E1B55K* and *E4orf*1 deleted Ad [13]. Interestingly, even while the late viral proteins and the transgene are expressed from the Ad MLP, cells infected by the different Ads produced detectable levels of the HIV transgene as measured by the levels of rhFLSC and gp120 protein (Figure 1D) in contrast to the Ad late proteins that were barely detectable in the Δ*E4^1−4^* Ad-infected cells. It may be that the levels of late viral proteins are affected by the loss of E1B55K [37] and E4orf3 [38], both of which have been implicated in the shuttling and/or accumulation of Ad late RNA. The rhFLSC transgene may not require these Ad proteins for efficient expression. This is speculative as we have not evaluated this.

### 3.2. E4orf1 Limits Intrinsic Innate Cytokine Gene Expression in ΔE1B Ad-Infected Cells

**Figure 2:**
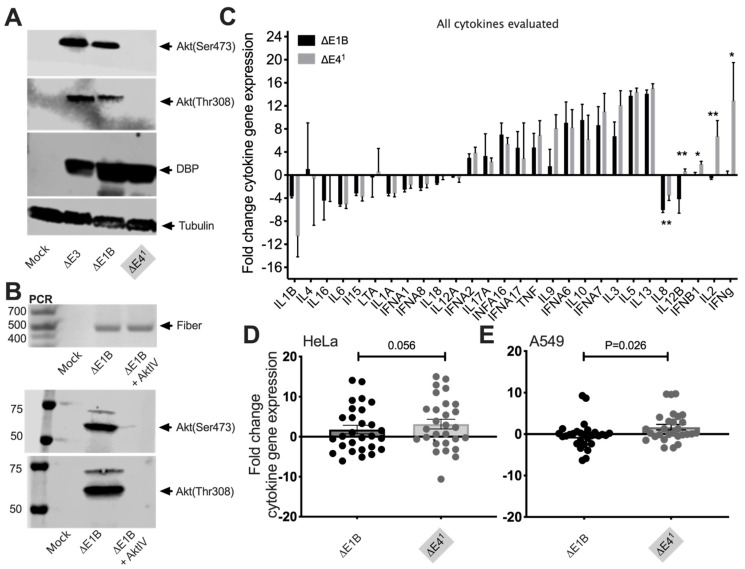
Effects of E4orf1 on innate cytokine gene expression in infected human epithelial cell lines. HeLa cells were infected at a MOI of 50 with Δ*E3,* Δ*E1B* and Δ*E4^1^* Ads and incubated for 48 h as described in Materials and Methods. (**A**,**B**) Western blotting using whole cell lysate shows the presence or absence of Akt phosphorylation at Ser473 and Thr308, with (**A**) Ad DNA binding protein (DBP) and (**B**) cells treated with or without AktIV. PCR for Ad fiber is used as the Ad infection control. (**C**) The fold change in expression levels of all the cytokines evaluated in HeLa cells are plotted. (**D**,**E**) The magnitudes of the induced/upregulated cytokines in each group are compared in (**D**) HeLa cells and (**E**) A549 cells. The differences in mean values are highlighted with colored boxes. For each cell line *n* = 2 biological repeats performed in triplicate. Mean plus SEM are plotted. *p* values obtained by Mann–Whitney unpaired *t*-test and Wilcoxon matched-pairs signed-rank test * = *p* < 0.05, ** = *p* < 0.01.

E4orf1 stimulates the PI3 Kinase/Akt pathway [13] that mediates immune responses in other cellular systems [14,39]. To investigate the possibility that E4orf1 signals affect innate cytokine gene expression, we infected HeLa cells at an MOI of 50 with Δ*E3* Ad and Δ*E1B* Ad that contained E4orf1 and Δ*E4^1^* Ad that did not (Figure 2). As shown in Figure 2A, in the ΔE3 and ΔE1B infected cells, Akt is phosphorylated on serine (Ser) 473 and threonine (Thr) 308, whereas it is not in Δ*E4^1^* infected cells. In Figure 2B, HeLa cells were infected with the Δ*E1B* Ad and treated 4 h later with or without AktIV, known to prevent the phosphorylation of Akt. The same antibodies used in Figure 2A did not recognize phosphorylated Akt in AktIV-treated Δ*E1B* Ad infected cells, confirming the antibody specificity and supporting the notion that cells infected with Δ*E3 or* Δ*E1B* Ad contain phosphorylated Akt (Ser473 and Thr308), in stark contrast to cells infected with Δ*E4^1^* Ad (Figure 2A, [13,17]). A PCR for the Ad fiber gene (Figure 2B) was used to confirm infection of the cells. In Figure 2C, we compare fold change in innate cytokine-related genes from Δ*E1B* (black bars) and Δ*E4^1^* (gray bars) Ad-infected HeLa cells. HeLa cells infected with Δ*E4^1^* Ad exhibited elevated levels of IFNB, IFNγ, IL12B, and IL2 (Figure 2C). To determine if there were any differences in the overall magnitude of cytokine genes that were either up- or downregulated, we replotted the mean of the individual values from Figure 2C using a scatter plot (Figure 2D). While no differences were observed in the magnitude of genes that were downregulated, the magnitude of genes that were induced upon infection with the Δ*E4^1^* Ad was higher than that induced in cells infected with the Δ*E1B* Ad (Figure 2D). To further confirm that the presence of E4orf1 affects innate cytokine gene expression in Δ*E1B* Ad-infected cells, we repeated the experiments in the lung epithelial A549 cells because Ad is known to infect via the upper respiratory tract. In A549 cells (Figure 2E), as in the HeLa cells (Figure 2D), no differences were observed in the magnitude of genes downregulated. By contrast, the magnitude of genes that were induced upon infection with the Δ*E4^1^* Ad were higher than those induced in cells infected with the Δ*E1B* Ad (Figure 2E). From these experiments it appears that E4orf1 may limit expression levels of innate cytokine genes in Δ*E1B* Ad-infected cells.

### 3.3. E4orf1 Limits Levels of IFNγ-Producing Memory T-Cells in Immunized Rhesus Macaques

**Figure 3:**
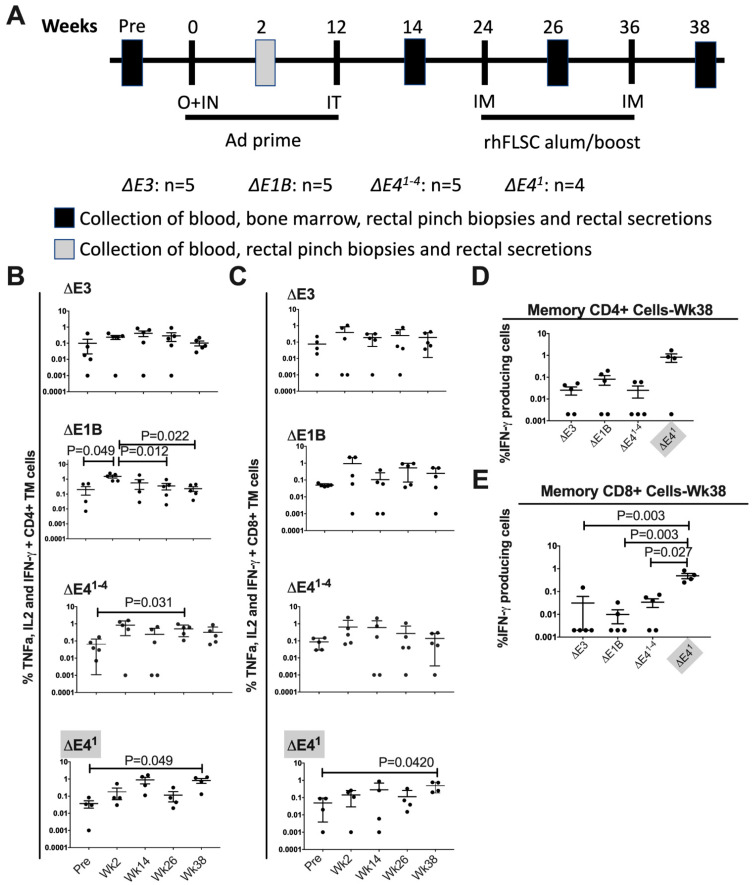
(**A**) Animal immunization and sample collection scheme. Macaque groups were immunized with the indicated Ad vectors all containing the rhFLSC transgene. O = oral, IN = intranasal, IT = intratracheal and IM = intramuscular. (**B**,**C**) The percentages of the combined levels of all three cytokines (IFNγ, TNFα and IL-2) that were expressed by memory CD4+ (panel B) and CD8+ (panel C) T-cells in PBMC from the rhesus macaques immunized with the indicated vectors are shown over the course of immunization. (**D**,**E**) The percentages of IFNγ cytokine-producing memory CD4+ and CD8+ T-cells in PBMC of rhesus macaques at wk 38 post-immunization. *p* values were obtained using one-way ANOVA with Tukey’s multiple comparisons Post Hoc test and for (**E**) were corrected by the method of Sidak for all the possible pairwise comparisons.

To further investigate the effects of E4orf1 and its deletion on immune responses induced by Δ*E1B* Ad, we immunized rhesus macaques with rhFLSC-expressing Ads, as detailed in Materials and Methods and outlined in Figure 3A. Immunization of Rhesus macaques with rhFLSC-expressing Δ*E3* Ad followed by boosting with rhFLSC protein in alum adjuvant was reported to elicit rhFLSC-specific cytokine-producing T-cells in PBMC [18]. Therefore, we measured the levels of T-cells that were producing IFNγ, TNFα and IL-2 in PBMCs by intracellular staining following stimulation with HIV_BaL_ gp120 pooled peptides (Figure 3). No significant changes in the fraction of CD4+ total memory (TM) cells were noted in Δ*E3* Ad-immunized macaques. Two weeks after the first prime, the levels of cytokine-producing TM CD4+ T-cells significantly increased in macaques immunized with Δ*E1B Ad* compared to pre-immunization values (Figure 3B). The high levels were only sustained in macaques immunized with the Δ*E4^1^*^−*4*^ and Δ*E4^1^* Ad (Figure 3B).

We evaluated the levels of TM CD8+ T-cells in the immunized macaques. The fraction of TM CD8+ T-cells producing all three cytokines increased two weeks after the first prime in all immunized groups, but not significantly (Figure 3C). However, at wk 38, post-immunization macaques immunized with the Δ*E4^1^* Ad exhibited significantly higher levels of TM CD8+ T-cells compared to the pre-timepoint (Figure 3C). The elevated memory T-cell responses at 38 weeks are important as they suggest the readiness of the immunized macaques to respond to an HIV exposure. The percentage of memory CD4+ T-cells that were producing IFNγ at 38 weeks appeared to be highest in the Δ*E4^1^* Ad-immunized group compared to the other immunization groups; however, because of the large variance, an ANOVA could not be performed (Figure 3D). With respect to the percentage of memory CD8+ T-cells, an ANOVA revealed significant differences between the Δ*E4^1^* and the other Ad-immunized groups (Figure 3E). Importantly, the Ad with the deletion of *E4orf1*, Δ*E4^1^* Ad, induced higher levels of specific memory CD8+ T-cells than the Ads that contain *E4orf1*: ΔE3 and Δ*E1B* Ad (Figure 3E).

**Figure 4:**
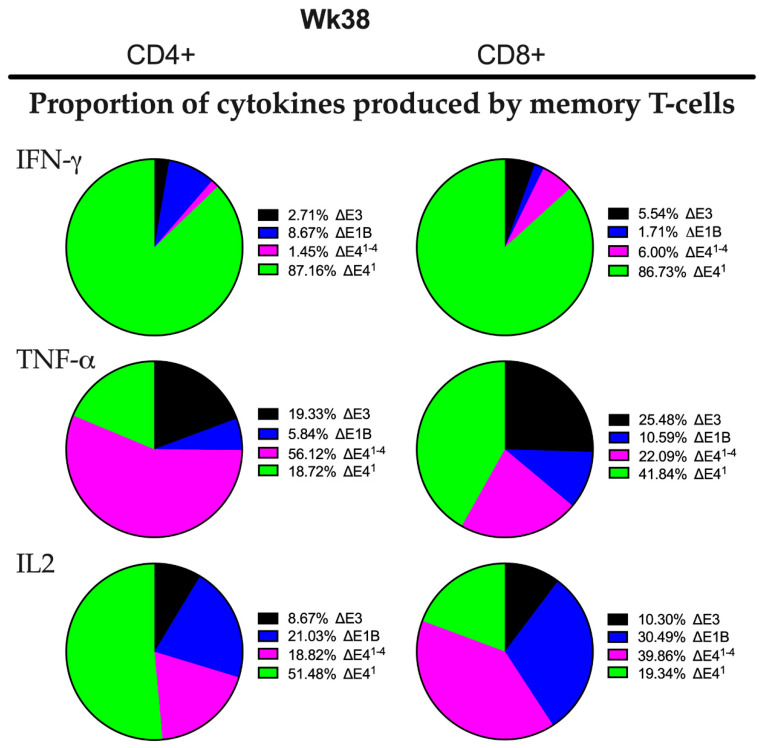
Pie chart comparison of the relative numbers of cytokine-producing memory T-cells for each Ad vector. The total numbers of cytokine-producing memory T-cells for each vector at wk 38 post-immunization were graphed as a proportion of the total number of cytokine-producing memory T-cells for each cytokine.

To better understand how Δ*E4^1^* Ad impacted the memory CD4+ and CD8+ T-cells, we plotted pie charts that display the proportion of IFNγ, TNFα and IL-2 that were induced by each Ad. All the Ads provoked the induction of varying levels of all three cytokines evaluated (Figure 4). Thus, while IFNγ was produced by the majority of CD4+ and CD8+ memory T-cells 38 weeks after immunization with Δ*E4^1^* Ad, a large portion of the CD4+ cells also produced IL-2 and a similar large portion of CD8+ cells produced TNFα (Figure 4). We did not carry out Boolean analysis, thus the cells in Figure 3 and Figure 4 may have expressed more than one cytokine. Nonetheless, the very visible increases in IFNγ-producing memory T-cells observed in the Δ*E4^1^* Ad-immunized rhesus macaques suggest that an activity associated with E4orf1 limits the levels of cytokine-producing memory CD4+ and CD8+ T-cells in Δ*E1B* Ad-immunized rhesus macaques.

### 3.4. Levels of HIV-Specific Memory B-Cells May Be Independent of E4orf1

**Figure 5:**
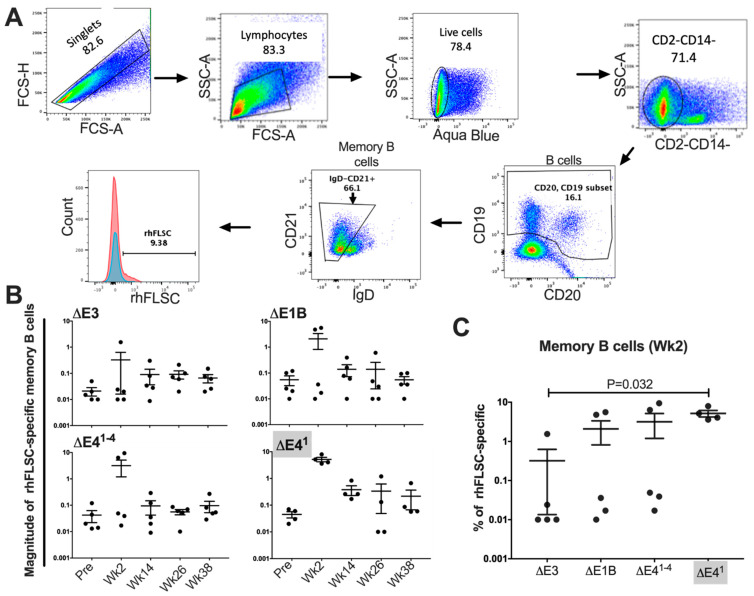
Mucosal rhFLSC-specific memory B-cell gating strategy and frequency in Ad vector-immunized rhesus macaques. (**A**) Representative gating of memory B-cells. (**B**) Percentages of rhFLSC-specific memory B-cells over the course of immunization were calculated based on total B-cells at each time point. *p* values were obtained using the Wilcoxon signed-rank test. (**C**) Percentage of rhFLSC–specific memory B-cells for each vector at Week 2 post-initial immunization. *p* values were obtained using the Mann–Whitney two-tailed test.

Memory B-cells induced by Ad5hr-HIV and SIV recombinants have been previously reported to correlate with functional antibody responses and reduced viremia [40] in a prime/boost regimen in rhesus macaques. Therefore, it was important to determine if and how the Ads used here affect specific memory B-cells in mucosal tissues where HIV is believed to make its entry [41]. For this, class-switched memory B-cells in rectal tissue were gated as previously reported [27,28], first for singlets, followed by lymphocytes. From the lymphocytic gate we separated out the live cells with the cell viability marker, Aqua (Invitrogen). We gated out the monocytes, selected the CD19- and CD20-positive B-cells, and from this population, determined the percentage of rhFLSC-specific memory B-cells (Figure 5A). We noted that the distribution of the data points for the memory B-cells two weeks post-initial immunization in rectal tissue (Figure 5B) was not like that of systemic T-cells in Figure 3. For example, compare the grouping of the data in the Δ*E1B* Ad-immunized macaques in Figure 5B to the same Δ*E1B* Ad-immunized group in Figure 3B. While total memory CD4+ T-cells increased significantly 2 weeks post-immunization, the memory B-cells did not. This is curious and may speak to differences in the development of memory CD4+ T-cells and B-cells in these animals. Overall, the magnitude of rhFLSC-specific memory B-cells two weeks post-initial immunization in rectal tissue of the ΔE1B, Δ*E4^1^*^−*4*^, and Δ*E4^1^* immunized macaques tended to increase, but compared to pre-levels, the increases never became significant and, in all cases, plateaued thereafter (Figure 5B). In Figure 5C, we compared the levels of memory B-cells present at two weeks following the initial immunization for each of the Ad-immunized groups of rhesus macaques. The Δ*E4^1^* Ad group exhibited a significantly higher percentage of transgene-specific memory B-cells compared to those of macaques infected with the Δ*E3* Ad (Figure 5C). These expanded memory B-cells that thereafter contracted may be short-lived memory B-cells made independent of germinal center sites [42,43]. These observations need to be confirmed in a larger cohort of animals but suggest that in Δ*E1B* Ad, the deletion of E4orf1 may impact memory B-cells.

### 3.5. E4orf1 Limits Levels of IgG1 Antibodies in Immunized Rhesus Macaques

**Figure 6:**
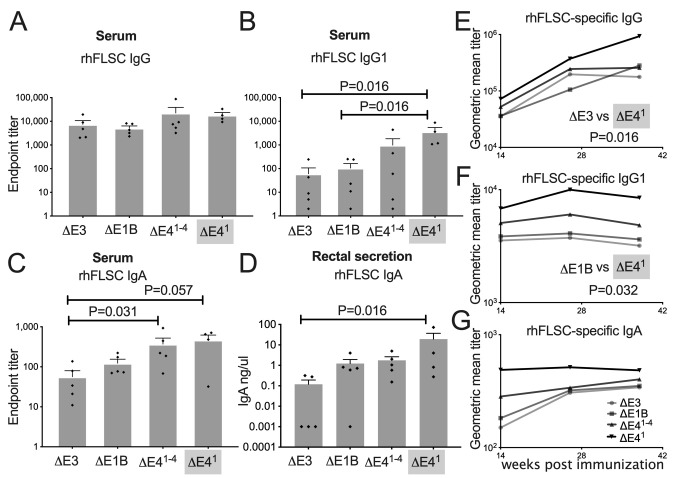
Levels of Env-specific serum and mucosal antibodies post-immunization in rhesus macaques. Titers of rhFLSC-specific IgG (**A**), IgG1 (**B**), and IgA (**C**) binding antibodies in sera at week two post-immunization. Significant differences in responses of IgG1 (panel B) and IgA (panel C) between vectors shown were obtained using the Mann–Whitney unpaired two-tailed test. (**D**) Quantity of rhFLSC-specific IgA binding antibody at week 14 post-immunization in rectal secretions. (**E**–**G**) Persistent induction of Env-specific antibodies in Ad vector-immunized rhesus macaques. (**E**) Titers of rhFLSC-specific binding IgG antibody at weeks 14, 26 and 38 post immunization in serum samples. (**F**,**G**) Titers of rhFLSC-specific IgG1 and IgA binding antibodies respectively in sera at weeks 14, 26 and 38 post-immunization. Statistically significant differences were obtained using the Mann–Whitney unpaired two-tailed test.

Ad5hr HIV-vaccine candidates are known to promote high levels of HIV-specific antibodies in rhesus macaques [18]. Recently, we reported that Ad *E1B55K* and the *E4orf1–4* gene-products suppress HIV-specific antibody levels in immunized macaques [12]. This limited the possible gene-products responsible for the suppression to E4orf1, E4orf2, E4orf3 and/or E4orf4. We hypothesized that Ad E4orf1 affects levels of HIV-specific binding antibodies in immunized macaques and assessed the levels of binding antibodies found in the blood and in rectal secretions. As shown in Figure 6A, the levels of rhFLSC-specific immunoglobulin (Ig) class G (IgG) at week 2 post-immunization were elevated in the serum of Δ*E4^1−4^* and Δ*E4^1^* Ad-immunized macaques, but not significantly so. In our recent study, the Δ*E4^1−4^* Ad-immunized macaques produced higher levels of IgG1 than Δ*E1B* and Δ*E3* Ad-immunized macaques [12]. This trend was observed here also (Figure 6B). While the mean level of IgG1 of the Δ*E4^1^* Ad-immunized macaques was not different from that of the Δ*E4^1−4^* Ad-immunized macaques, it was significantly elevated at week 2 post-immunization compared to levels of IgG1 induced in the Δ*E1B* and Δ*E3* Ad-immunized macaques (*p* = 0.016 for each) (Figure 6B). These results suggested that an activity associated with E4orf1 influences the levels of IgG1 in Ad-immunized macaques.

Immunoglobulin class A, IgA, predominates in mucosal compartments and exists second to IgG systemically [44,45]. However, it is not clear if IgA is protective against HIV. Passive administration of IgA antibodies protects uninfected hosts from HIV and related primate immunodeficiency viruses [46]. However, in RV144, plasma IgA specific for the C1 region of the HIV envelope blocked effector function of IgG [47]. Nonetheless, we measured the levels of rhFLSC-specific IgA in the serum and rectal secretions of the immunized macaques (Figure 6C,D). rhFLSC-specific IgA binding antibody at wk 2 post-immunization in the serum of macaques immunized with Δ*E4^1−4^* was significantly higher than levels in serum of macaques immunized with the Δ*E3* Ad (*p* = 0.031) (Figure 6C). Levels of rhFLSC-specific IgA binding antibody in serum of macaques immunized with the Δ*E4^1^* Ad were also enhanced (*p* = 0.057) compared to levels in serum of macaques immunized with the Δ*E3* Ad (Figure 6C). The Δ*E4^1^* Ad-immunized macaques also produced significantly higher rhFLSC-specific IgA in rectal secretions at wk 14 post-immunization compared to the Δ*E3* vector (*p* = 0.016; Figure 6D). The antibody titers illustrated in Figure 6A–D were obtained before the animals were boosted with rhFLSC protein. This demonstrates that all four Ads induced specific IgG, IgG1 and IgA in immunized Rhesus macaques. Thirty-six weeks after the initial immunization the geometric mean of the serum IgG titers was significantly elevated (*p* = 0.016) in the Δ*E4^1^* Ad-immunized rhesus macaques compared to the Δ*E3* Ad-immunized macaques (Figure 6E). As in Figure 6B, the serum IgG1 titers at 38 weeks were highest in Δ*E4^1^* Ad-immunized rhesus macaques compared to the Δ*E1B* Ad-immunized macaques (*p* = 0.032; Figure 6F). In Figure 6G, the same trend was observed where serum IgA titers were highest in Δ*E4^1^* Ad-immunized rhesus macaques compared to the other Ad-immunized macaques, but not significantly. Thus, differences in antibody titers that were observed early in the Δ*E4^1^* Ad-immunized rhesus macaques were enhanced by the rhFLSC boost. Importantly, in Figure 6B,F, animals immunized with the Ad in which *E4orf1* was deleted, Δ*E4^1^* Ad, consistently produced higher levels of IgG1, than the parental Δ*E1B* Ad. These results suggest that an activity associated with E4orf1 limits levels of IgG1 produced in Δ*E1B* Ad-immunized rhesus macaques.

### 3.6. Levels of HIV-Specific Neutralizing Antibody Titers May Be Independent of E4orf1

**Figure 7:**
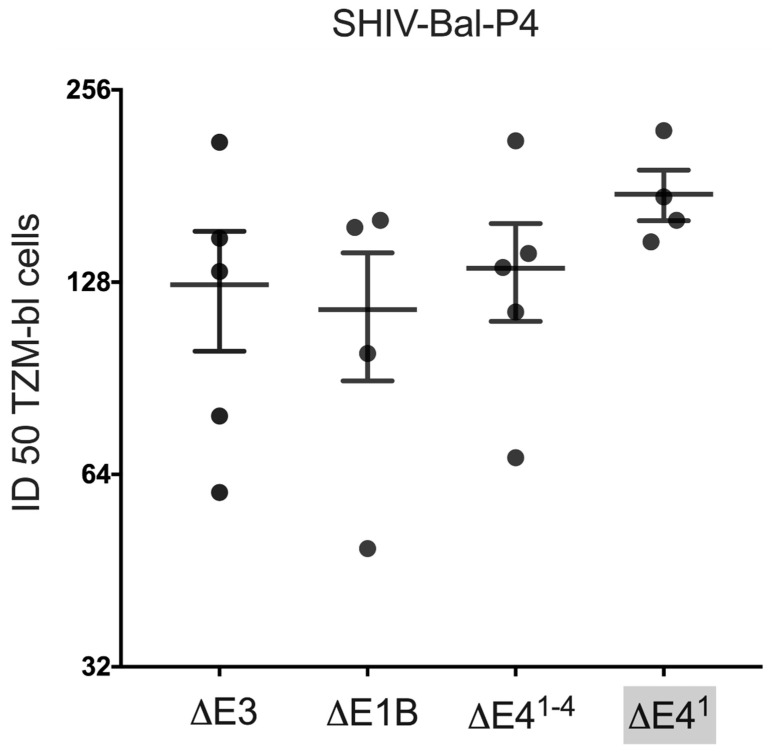
Neutralizing antibody titers in Ad vector immunized rhesus macaques. The neutralization levels of antibodies against SHIV Bal-P4 for the different vectors were assessed at wk 38, as described in Materials and Methods.

Neutralization is the one function that most describes effective antibodies. We have reported that Ads expressing rhFLSC promote high levels of neutralizing antibodies against SHIV Bal-P4 [12,18] and other HIV strains [18]. Therefore, it was important to learn if E4orf1 affects levels of neutralizing antibodies produced in Ad-immunized rhesus macaques. We measured the neutralization titers in serum at wk 38. The SHIV Bal-P4 neutralization antibody titers for the Ad-immunized macaques in all groups were similar (Figure 7), although the geometric mean neutralizing titer was highest in Δ*E4^1^* Ad-immunized rhesus macaques. Thus, deletion of E4orf1 from the Δ*E1B* Ad did not affect specific antibody neutralization levels in immunized rhesus macaques.

### 3.7. E4orf1 Limits ADCC Outcome in Immunized Rhesus Macaques

**Figure 8:**
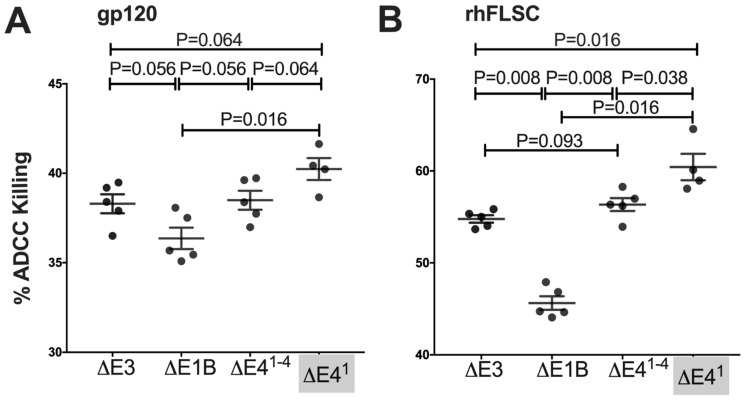
ADCC activity in Ad vector-immunized rhesus macaques. ADCC activity was measured at wk 38 (2 wks post second protein boost) using plasma from immunized rhesus macaques. Antibodies were evaluated against HIV-1Bal gp120 and rhFLSC-coated EGFP-CEM-NKr-CCR5-SNAP cells as targets as described in Materials and Methods. (**A**,**B**) percent ADCC killing against HIV-1Bal gp120 and rhFLSC, respectively. *p* values obtained using the Mann–Whitney unpaired two-tailed test.

It is widely accepted that vaccine-mediated protection should elicit polyfunctional immune responses, including Fc-mediated antibody-dependent cellular cytotoxicity (ADCC). This was recently highlighted in the RV144 clinical trial where ADCC activity correlated with protection in the absence of IgA responses [48,49]. Accordingly, we investigated whether E4orf1 impacted ADCC in immunized rhesus macaques. HIV-1_Bal_ gp120 or rhFLSC target cells were used to detect ADCC activity two weeks after the second boost, as described in Materials and Methods. All sera from the immunized macaques produced measurable ADCC killing, and as with most of the immune responses measured so far, here too the Δ*E4^1^* Ad-immunized macaques exhibited greater percent killing compared to the other groups of immunized macaques (Figure 8). The Δ*E4^1−4^* Ad-immunized rhesus macaques exhibited a marginally higher mean percent killing of gp120-specific ADCC mediating antibodies than the Δ*E1B* Ad-immunized rhesus macaques (*p* = 0.056, Figure 8A). The difference was even more pronounced when gp120 target cells were replaced with rhFLSC targets (*p* = 0.008, Figure 8B). In fact, the percent killing of ADCC antibodies using rhFLSC target cells was elevated for all the Ad-immunized macaque groups compared to the percent killing observed using gp120 targets (Figure 8A,B). Interestingly, using gp120 target cells, a significant difference was observed for the mean ADCC percent killing of Δ*E4^1^* Ad-immunized macaques compared to that of the ΔE1B Ad-immunized macaques (*p* = 0.016, Figure 8A). Since when either gp120 (Figure 8A) or rhFLSC targets (Figure 8B) were used, the Δ*E4^1^* Ad-immunized rhesus macaques exhibited significantly higher levels of ADCC killing than the Δ*E1B Ad-*immunized rhesus macaques (*p* = 0.016 for both), we conclude that Ad E4orf1 limits antibodies that mediate ADCC in Δ*E1B Ad-*immunized rhesus macaques.

## 4. Discussion

Recently, we showed in mice and rhesus macaques that Δ*E4^1−4^* Ad induces higher HIV-specific binding antibody and cytokine-producing memory T-cells compared to Δ*E3* Ad [12]. This suggested that either E4orf1, E4orf2, E4orf3 and/or E4orf4 mediates immune responses. Here we focused on E4orf1 because it has been reported to stimulate signals through the PI3K/Akt pathway [13,50] that mediates immune responses [14]. While we did not link Akt directly to the adaptive immune responses observed in the immunized rhesus macaques, nor to the cytokine genes that were differentially expressed in the *E4orf1*-deleted Δ*E4^1^* Ad-infected cells, we nonetheless showed in Figure 2 that levels of phosphorylated Akt in the *E4orf1*-containing Δ*E1B* Ad-infected cells were reduced in *E4orf1*-deleted Δ*E4^1^* Ad-infected cells. Furthermore, the cells with little or no phosphorylated Akt induced elevated levels of several cytokine genes compared to Δ*E1B* Ad-infected cells that stimulated the phosphorylation of Akt. In other cell systems, Akt allows the activation of mTOR1 that mediates the phosphorylation activation of NF-kB [51]. In a recent publication we provide further evidence supporting a link between E4orf1 and activation of NF-kB [17] which is known for its involvement in the induction of innate cytokine genes important for stimulating both innate and adaptive arms of the immune system [16]. Moreover, we showed that *E4orf1*-deleted Δ*E4^1^* Ad-immunized rhesus macaques produced higher levels of IFNγ-producing memory T-cells (Figure 4), higher levels of transgene-specific IgG1 (Figure 6), and antibodies able to mediate greater ADCC activity (Figure 8) than the *E4orf1*-containting Δ*E1B* parental Ad. Thus, our results indicate that the effects of E4orf1 on immune responses are repressive in nature.

Vaccine-elicited cellular responses can control viremia in rhesus macaques [52]. Additionally, T-cells are necessary for sustaining memory B-cell expansion [53]. In this study, we compared immune responses induced by *E4orf1*-containing and *E4orf1*-deleted Δ*E1B* Ad HIV vaccine candidates. All the Ads except the ΔE3 Ad induced enhanced HIV-specific TNFα, IL2, and IFNγ-producing memory CD4+ T-cells at one time or another over the course of immunization (Figure 3B). Moreover, 38 weeks following the initial immunization with the Δ*E4^1^* Ad, most of the CD4+ T-cells produced IFNγ (Figure 4). However, this, as we mentioned (Figure 3D) was not significant because of the large variance. It has been well established that IFNγ from CD4+ T-cells protects against infections [54,55] and early, strong type I and II IFN responses can promote the development of functional antibody responses and CD4+ T-cell responses to vaccine antigens [56]. This is because IFNγ induces the production of proinflammatory cytokines and chemokines [57] and increases antigen presentation by macrophages and dendritic cells [58], as well as inducing class switching in B-cells [59]. However, IFNγ may enhance HIV-1 infection [60], a finding that stands in conflict with preclinical vaccine trials in macaques, where the durable and sustained suppression of viral load and lack of disease progression were positively associated with increased SIV-specific IFNγ CD4+ [61] and CD8+ [61,62,63] T-cell responses. These contrasting effects of IFNγ have complicated its therapeutic value for HIV [64]. Despite these observations, IFNγ is used as a biomarker for HIV antigenic responses [64].

Antigen-specific memory B-cells in vaccinated rhesus macaques [40,65] have correlated with ADCC activity. Thus, vaccines that increase specific memory B-cells may be desirable. Here, two weeks after the 1st prime, we observed a transient increase in memory B-cells in the rectal tissue of all the immunized rhesus macaques (Figure 5). These increases were not significant, except in the Δ*E4^1^* Ad-immunized rhesus macaques where levels of transgene-specific memory B-cells were higher than those in Δ*E3* Ad-immunized macaques at wk 2 post-immunization (Figure 5C). This increase was not sustained, however. Thus, overall, levels of memory B-cells were not obviously affected by E4orf1.

ADCC activity may be important for an HIV vaccine. ADCC elicited by vaccination correlates with reduced acute SIV viremia [66,67], and with reduced acute as well as chronic SHIV viremia. In HIV-infected people, ADCC activity associates with slower disease progression and reduced viremia [68,69,70,71,72,73]. Most recently, in the RV144 HIV vaccine efficacy clinical trial, ADCC activity correlated with protection [48,49]. We showed here that all the Ads used induced antibodies that mediated HIV-specific ADCC killing (Figure 8). Notably, the Δ*E4^1^* Ad induced antibodies that mediated greater ADCC activity and were able to kill a greater percentage of target cells (Figure 8). This is the first demonstration that Ad E4orf1 inhibits ADCC activity in immunized rhesus macaques.

The fact that results with the Δ*E4^1^* and Δ*E4^1−4^* viruses often differ suggests that the effects observed may be much more complex than our simplistic interpretations. Of the E4 products in the Δ*E4^1^* Ad, E4orf3 is reported to inactivate type 1 and type 2 interferon responses [10]. We showed recently that E4orf1 cooperates with E4orf3 to allow Ad-infected cells to survive with DNA > 4n which required NF-kB [17]. Thus, in Δ*E1B* Ad-infected cells, E4orf3 may cooperate with E4orf1 in suppressing immune responses. Besides E4orf3, E4orf4 regulates E1A activity, the E4 promoter, and activation of the Ad E2 promoter which controls viral DNA synthesis and induces apoptosis. Both E4orf1 and E4orf4 affect cap-dependent translation, S-phase entry and viral progeny production in normal and growth-limiting conditions [74]. In primary quiescent epithelial cells (SAEC), however, E4orf4 was shown to be more adept at activating mTOR than E4orf1 [75]. mTORC1 phosphorylation of the S6 kinase 1 (S6K1) and eukaryotic translation initiation factor 4E (eIF4E) binding protein (4EBP) facilitates 5′ cap-dependent mRNA translation [76]. In one experiment, even while the pattern of p70 S6K phosphorylation was altered relative to when E4orf1 was present in the Ad-infected cells, it remained detectable in *E4orf1*-deleted Ad-infected cells [13]. It is possible that the resulting phosphorylated p70 S6K may have been due to E4orf4, in which case E4orf4 may support translation of proteins that affect immune responses. Thus, in *E4orf4*-containing Ad E4orf4 may mediate immune responses. Some of the reported effects of E4orf4 may partially explain why in Figure 2C, the *E4orf1*-containing Ad-infected cells continue to express some innate cytokine-related genes at similar levels to those produced by *E4orf1*-deleted, Ad-infected cells, and why results with the Δ*E4^1^* that has *E4orf4* often differ from those with the Δ*E4^1−4^* Ad. Needless to say, the roles that the other E4 gene-products play in the Ad-induced immune responses remain to be formally evaluated. Nevertheless, evidence of effects of E4orf1 on immune responses was clearly shown by its deletion in the Δ*E4^1^*Ad that led to the induction of elevated levels of cytokines in cells and production of IFN-γ by CD8+ T-cells, higher levels of HIV-specific IgG1 and IgA, and greater ADCC activity in immunized rhesus macaques.

Finally, the *E1B55K*-deleted Ads that we used here are different from the *E1/E3*-deleted forms of Ad that are used in many vaccines such as the Johnson and Johnson (Ad26) [77,78], AstraZeneca (ChAd) [79,80], Sputnik V (Ad5 and Ad26) [81,82] and Convidicea (Ad5) [83] COVID 19 vaccines. The Ad5 we use is widely distributed among the general population. As such, pre-existing immunity may reduce its immunogenicity and, as a result, vaccine efficacy. The more exotic ChAd and less prevalent Ad26 provide a means to circumvent the potential preexisting immunity to Ad5. Serotype differences aside, perhaps an even greater difference between these Ads is E1A that is expressed in our Ads (Figure 1A) and not in the *E1/E3*-deleted forms [84]. E1A induces the activation of the other Ad early genes (E1B, E2, E3, and E4) [85]. As such, Ads that contain E1A maintain the ability to replicate where Δ*E1* Ads do not. The ability to replicate may offer significant advantages. For one, relative to the replication incompetent Δ*E1* Ads, replication competent Ads exhibit enhanced immune responses [4], in part due to longer presentation of the specific immunogens, and broad biodistribution [86]. Notwithstanding, the genes in the replication competent Ads are active, and as we have shown, the E4 gene products contribute to the quality and levels of the immune responses induced against the inserted transgene [12]. By contrast, because Δ*E1* Ads do not contain *E1A*, the *E4* genes in these Ads are considered to be inactive. However, in one study it was shown that deletion of the Ad E4 genes blunted host immune responses induced by Δ*E1* Ad [87]. Thus, E4orf1 (the focus of this article) may be active in all Ad vectors. Accordingly, our finding that E4orf1 suppresses immune responses may be relevant to all Ad vectors being explored for clinical use.

## Figures and Tables

**Table 1:** Antibodies used for T-cell intracellular staining.

Ab	Fluorochrome	Clone	Catalog #	Company
CD3	Alexa Fluor^®^ 700	SP34-2	557917	BD
CD4	PE	L200	550630	BD
CD 95	PE-cy5	DX2 (RUO)	559773	BD
CD28	PE-cy7	CD28.2	25-0289-42	eBiosciences
IFN-γ	FITC	B27 (RUO)	552887	BD
Il-2	APC	MQ1-17H12	551383	BD
TNFa	Brilliant Violet 785TM	MAb11	502948	Biolegend
CD8a	APC/Cy7	RPA-T8	301016	Biolegend
CD49d	Purified NA/LE Mouse Anti-Human	9F10 (RUO)	555501	BD
CD69	PE-CF594	FN50	562617	BD
CD107a	Brilliant Violet 711TM	H4A3	328640	Biolegend

**Table 2:** Antibodies used for B-cell Flow Cytometry.

Antigen	Fluorochrome	Clone	Catalog #	Supplier
CD2	Qdot605	S5.5	Q10172	Invitrogen
CD14	Qdot605	Tu14	Q10013	Invitrogen
CD19	PeCy5	J3-119	IM2643U	Beckman Counter
CD20	BV650	2H7	563779	BD Bioscience
CD21	PeCy7	B-ly4	561374	BD Bioscience
CD27	PerCP-eF710	O323	46-0279-42	eBioscience
∗ CD138	PE	DL-101	352306	Biolegend
IgD	Texas Red	Polyclonal	2030-07	Southern Biotech
IgG	APC-Cy7	G18-145	561297	BD Bioscience
Ki-67	Ax70	B56	561277	BD Bioscience
IFR-4	FITC	3E4	11-9858-82	Invitrogen
HLA-DR	BV421	Tu36	MHLDR28	Invitrogen
∗ Bcl-2	PE	Bcl-2/100	340576	BD Bioscience
rhFLSC	APC-Streptavidin	NA	NA	
Viability Dye	Aqua	NA	L34966	Invitrogen

## Data Availability

Not applicable.
